# SNOMEDtxt: Natural Language Generation from SNOMED Ontology

**DOI:** 10.3233/SHTI190429

**Published:** 2019-08-21

**Authors:** Olga Lyudovyk, Chunhua Weng

**Affiliations:** a Department of Biomedical Informatics, Columbia University, New York, NY, USA

**Keywords:** Systematized Nomenclature of Medicine, Natural Language Processing, Access to Information

## Abstract

SNOMED Clinical Terms (SNOMED CT) defines over 70,000 diseases, including many rare ones. Meanwhile, descriptions of rare conditions are missing from online educational resources. SNOMEDtxt converts ontological concept definitions and relations contained in SNOMED CT into narrative disease descriptions using Natural Language Generation techniques. Generated text is evaluated using both computational methods and clinician and lay user feedback. User evaluations indicate that lay people prefer generated text to the original SNOMED content, find it more informative, and understand it significantly better. This method promises to improve access to clinical knowledge for patients and the medical community and to assist in ontology auditing through natural language descriptions.

## Introduction

**Figure 1 –:**
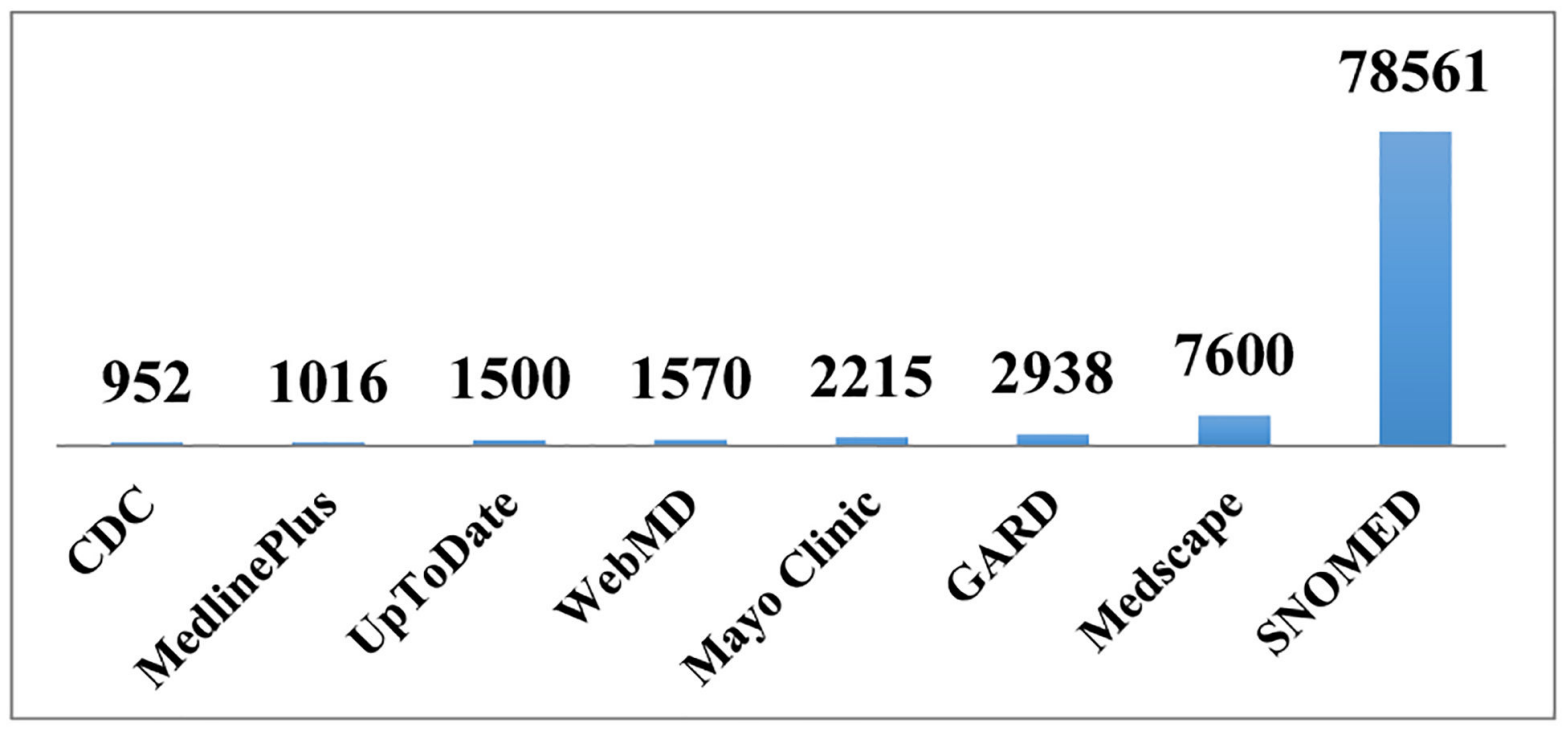
Counts of Diseases in cdc.gov, medlineplus.gov, uptodate.com, webmd.com, mayoclinic.org, rarediseases.info.nih.gov, medscape.com, SNOMED [2] (Nov. 10, 2018)

SNOMED CT is the world’s most comprehensive clinical terminology [1]. The March 2018 release of the US version contains 347,231 unique concepts, including 78,561 diseases, and defines 1,088,068 unique active relationships between these concepts [2]. In contrast, the largest professional medical reference source, Medscape (medscape.com), contains 7,600 diseases, representing less than 10% of the diseases defined in SNOMED CT, and the largest consumer health resource, Mayo Clinic (mayoclinic.org), describes 2,215 diseases. Disease descriptions in these resources are manually curated, thus limiting the number of diseases which can be covered. Topics may be chosen according to popularity in search results [3], thus rare diseases are often excluded from these resources. Counts of disease concepts in major medical information sources are shown in Figure 1. Google Knowledge Graph for diseases is not available, but since it is curated from the sources listed in Figure 1, it is likely on the same order of magnitude.

While extensive, SNOMED CT is not easily accessible to the public and is known to be difficult to use even for clinicians without training in ontologies [4,5]. Like other structured ontologies, SNOMED CT is not designed to be used directly by lay people. The US version of SNOMED CT contains only 4,372 text definitions easily interpretable by untrained personnel, covering 2,608 diseases, corresponding to 1.3% of all SNOMED CT concepts and 3.3% of disease concepts.

We propose a method called SNOMEDtxt to automatically generate disease descriptions from SNOMED CT in order to make available to both patients and the medical community the valuable clinical knowledge contained in SNOMED CT.

An additional use case for SNOMEDtxt is to enable clinicians and domain experts without specialised technical training or experience working with structured terminologies to review and critique clinical knowledge defined in SNOMED CT. This task is critically important as biomedical knowledge is growing exponentially with numerous data types and tools emerging rapidly on the daily basis. For example, Campbell et al. reported that the absence of a robust granular ontology represents a barrier to capturing and analyzing data in the field of cancer research and precision medicine [6], while Fung et al. made a similar observation in the area of rare diseases [7]. However, ontology auditing or quality ascertainment is largely performed by knowledge engineers with specialized training in ontology design and maintenance. The workforce of this profession is rare, hence creating a bottleneck for enabling scalable ontology expansion or for crowdsourcing ontology auditing. SNOMEDtxt allows representation of concepts and related information in natural text, thus expanding the group of potential reviewers to include any medical professionals who are not necessarily familiar with structured ontologies. Wider review of SNOMED CT by clinicians can be expected to improve accuracy, reduce missing information, and enable faster SNOMED CT evolution as the body of clinical knowledge expands.

Natural Language Generation (NLG) is a technology utilizing advanced computational methods to generate natural language descriptions from structured knowledge or data representation. Attempts to apply NLG to generate text from SNOMED CT have been reported by Liang et al. [8] and Kanhov et al. [9]. Liang and colleagues developed OntoVerbal, a generic tool for ontology verbalization that was then applied to SNOMED CT. While Kanhov and colleagues utilized an off-the-shelf natural language generator, they developed a methodology for user evaluation of the fluidity and readability of NLG texts in the Biomedical domain. OntoVerbal was developed as a Protégé 4.2 plugin and is not available in more recent Protégé versions or as a standalone application. The NLG system developed by Kanhov et al. was not made available for download or use.

OntoVerbal implements a generic verbalization approach for ontologies, with an emphasis on the ability to handle any OWL ontology and generate natural language descriptions for any entity type in that ontology [8]. This approach restricts handling of relationships, or ontology axioms, to generic lexical choices and results in some redundant and inelegant phrases, such as “*chronic disease of the genitourinary system* … *has a finding site in a structure of the genitourinary system*.” In contrast, our method trades off generalizability for improved readability and comprehensibility through more specific verbalizations of SNOMED CT axioms and simplifying structures tailored to SNOMED CT concepts, so that the same construct is simplified by SNOMEDtxt as “… *affects the genitourinary system*.” Moreover, OntoVerbal takes the generic approach to ordering information from simpler sentences to more complex ones, whereas SNOMEDtxt follows the common flow of information found in disease descriptions in reference medical texts: definition is followed by possible causes, presentation, diagnosis, clinical course, and finally additional information.

SNOMEDtxt is a novel NLG engine and interface, intended to evolve and improve over time with user feedback. The current version focuses specifically on disease concepts and can be easily extended to summarize procedures, treatments, and other information contained within SNOMED CT and relevant to the wider audience.

## Methods

**Figure 2 –:**
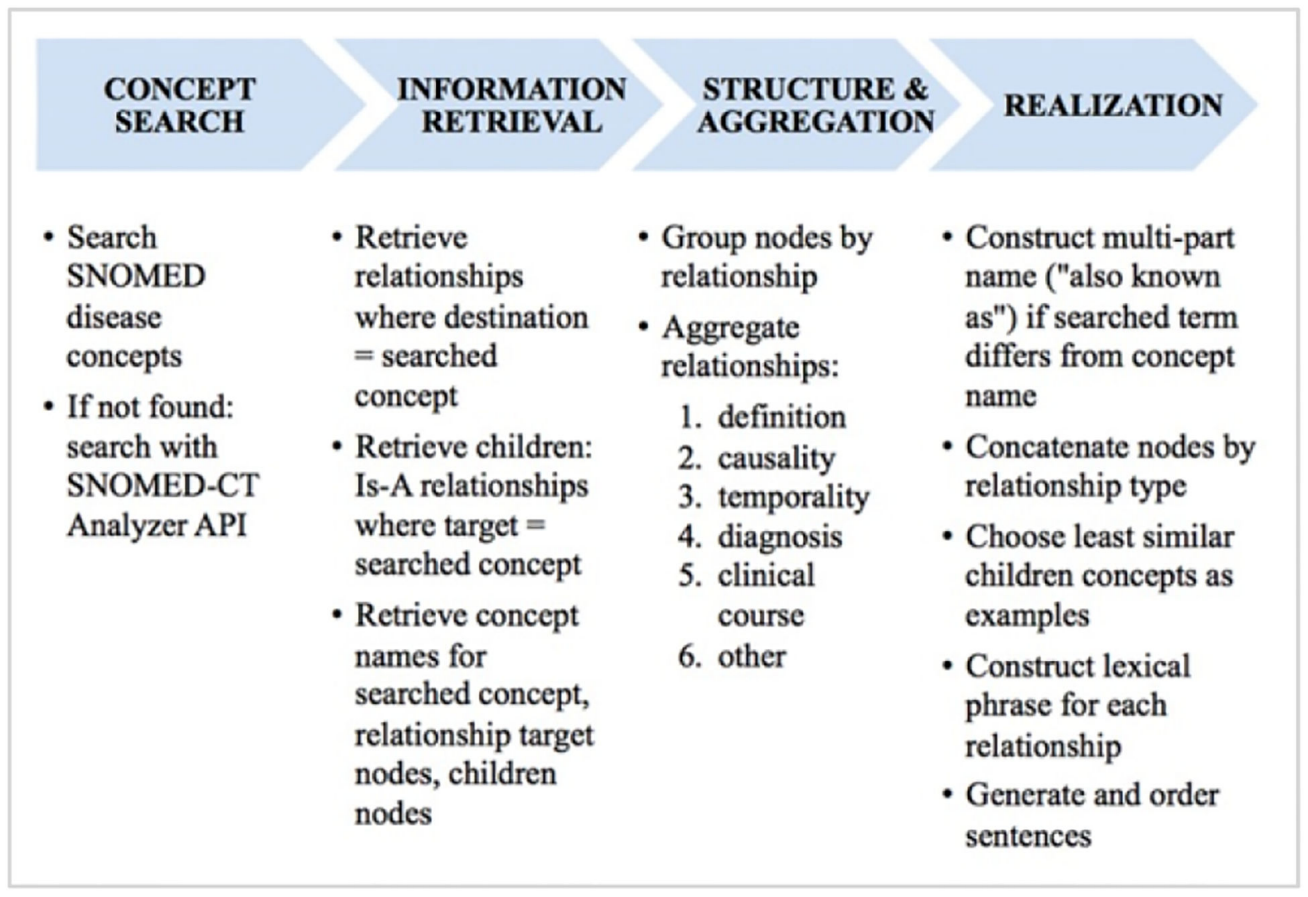
Framework for Disease Description Generation

SNOMEDtxt follows a 4-step framework outlined in Figure 2 to generate a disease description for a given disease.

### Concept Search And Information Retrieval

The current implementation of SNOMEDtxt is based on the 03/01/2018 release of SNOMED CT terminology, US edition, Snapshot version, available for download from the SNOMED CT website [2]. SNOMEDtxt uses a local copy of this database.

The system has the capability to randomly sample diseases from SNOMED CT and to search for disease names entered by a user. The search is undertaken in two steps: first, a simple match on concept names and synonyms in SNOMED CT database is attempted. If the search term is not found, the system then uses SNOMED CT Analyzer API (snomedct.t3as. org) to search for the term, provided that the API site is online.

Concepts are the key component of SNOMED CT. They are organized in a polyhierarchical structure with “Is-A” (parent-child) relationship and can be additionally defined or described through other relationships. Each relationship has a type, a source concept, and a destination concept. Once a disease Concept ID is found, relevant relationships are retrieved from SNOMED CT database:

- Relationships where the searched disease Concept ID is the source
- Is-A relationships where the searched disease Concept ID is the destination: the source concepts represent subtypes or examples of the disease and are included in the definition

Concept names are then retrieved for the corresponding target concepts. The generated text is the product of concept names arranged in lexical patterns corresponding to types of relationship between these concepts. Concept names undergo minimal string cleaning to remove non-informative structures such as “(Disorder)” and “(Body Structure)”.

### Structure And Aggregation

In order to produce fluid and coherent text and avoid redundancy wherever possible, SNOMEDtxt aggregates and structures information in three steps: Firstly, it groups all target nodes for the same relationship; secondly, it organizes relationships in broad logical groups; thirdly, it orders relationships within each group and the groups themselves following a typical flow of information in a disease description in medical reference texts. This stepwise grouping of relationships is a simplified application of the Rhetorical Structure Theory [10] that describes a recursive approach to organizing relationships in a text.

### Text Realization

The first task SNOMEDtxt undertakes in the Text Realization phase is constructing an informative disease name. If the search term is significantly different from the preferred term for the disease concept, as measured by Jaro-Winkler string distance [11], the disease description will combine both in the form of “<Preferred disease concept name> (also known as <searched term>)”, e.g. “Influenza (also known as flu)”.

Additionally, SNOMEDtxt concatenates all target nodes for the same relationship type which were aggregated in the previous step by following the “A and B”, “A, B, and C” format. When concatenating examples of a given disease, SNOMEDtxt selects a maximum of three examples, based on the largest string dissimilarity with the given disease name, as a tradeoff between completeness and relevancy.

Finally, relationship types are converted into corresponding lexical patterns (see Table 1) and sentences are generated. For the sake of conciseness, relationships in the same group are combined into one sentence wherever this approach produces fluid text. For example, the Is-A and the Finding site relationships are combined into one sentence that forms the concise definition of the disease: “*Asthma is a kind of Respiratory disorder that affects the Airway*”. Sentences are then ordered according to the order of relationships in Table 1.

## Results

### User Interface of SNOMEDtxt

A simple user interface is implemented in RShiny and is available online at https://sno2eng.shinyapps.io/sno2Eng.

An example disease description generated by SNOMEDtxt and the corresponding concatenated SNOMED CT content are illustrated below.

#### SNOMEDtxt Disease Description

Lupus erythematosus (also known as Lupus) is a kind of Autoimmune disease and Connective tissue disease that affects Connective tissue. Some examples of Lupus erythematosus are Systemic lupus erythematosus, Drug-induced lupus erythematosus, and Neonatal lupus erythematosus. Pathological process associated with Lupus erythematosus is AI - autoimmune. Other related concepts are Cutaneous lupus erythematosus, Lupus erythematosus profundus, and Discoid lupus erythematosus of eyelid.

#### SNOMED CT Content

ConceptID: 200936003. Terms: Lupus erythematosus, LE - Lupus erythematosus, Lupus, Lupus erythematosus (disorder). Relationships: Disorder of connective tissue (disorder) = Is a (attribute). Connective tissue structure (body structure) = Finding site (attribute). Autoimmune disease (disorder) = Is a (attribute). Autoimmune (qualifier value) = Pathological process (attribute). Related concepts: Systemic lupus erythematosus (disorder) - Is a (attribute). Drug-induced lupus erythematosus (disorder) - Is a (attribute). Neonatal lupus erythematosus (disorder) - Is a (attribute). Discoid lupus erythematosus (disorder) - Due to (attribute).

We evaluated disease descriptions generated by SNOMEDtxt against the concatenated SNOMED CT content using computed metrics and user evaluations. Both sets of evaluations indicate that SNOMEDtxt succeeds in making SNOMED CT content more readable and comprehensible.

### Computed Metrics

We computed readability and redundancy metrics for disease definitions of the top 20 most searched diseases in 2017 [13] and of 20 diseases randomly retrieved from SNOMED CT:

1. Readability: Flesch-Kincaid grade level (FK) and Automated Readability Index (ARI) estimate the number of years of education needed to understand a text. We calculated both with sylcount R package [12].
2. Redundancy: calculated as the ratio of unique word count to total word count after removing stop words.

Full summaries of health concepts retrieved from Medline Plus web service (MedlinePlus.org) were used as reference for the first set of disease concepts. Since only 4 out of 20 randomly sampled disease concepts had a reference health topic in Medline Plus, comparison with reference is not provided for the second set.

For both measures of readability, a lower score indicates a lower grade of education needed to understand the text and therefore better readability. These metrics indicate that SNOMEDtxt texts are more readable than the original SNOMED CT content. For the 20 most searched diseases, the average FK score for SNOMEDtxt texts (14.3) is equivalent to the second year of undergraduate degree, and FK for SNOMED CT content (17.9) corresponds to the graduate school level. ARI score of 12.0 for SNOMEDtxt is equivalent to twelfth grade, while ARI of 15.0 for SNOMED CT content indicates that the text is appropriate for readers at the Professor level. Readability scores for the MedlinePlus reference texts are significantly lower, indicating that they can be read by a much wider audience than either SNOMEDtxt or the original SNOMED CT content.

SNOMEDtxt texts also improve on the redundancy metric compared to SNOMED CT content for the top 20 searched diseases (0.74 vs. 0.55) and for the 20 randomly sampled diseases (0.69 vs. 0.56).

### User Evaluation

A survey evaluating results of SNOMEDtxt was conducted among 51 lay people recruited using Amazon Mechanical Turk (MTurk) and 6 clinicians from Columbia University Medical Center. MTurk is a crowdsourcing marketplace that enables outsourcing tasks like surveys to a distributed workforce for a small reward. Evaluations of all MTurk taskers that applied and did not self-identify as clinicians were included in the results. Evaluations of all 6 clinicians who responded to the survey were included in the results. All evaluators were provided with a basic description of the project, but were not aware of the study design or the research question.

We randomly selected a set of 20 disease concepts from SNOMED CT for evaluating readability, preference, accuracy, and completeness (set 1). Helpfulness was evaluated on a set of 20 disease concepts for which a medical reference text was available (set 2). Questions probing the degree of understanding were constructed for 10 diseases with sufficient information selected from 40 randomly sampled disease concepts (set 3). Comparison with OntoVerbal was restricted to 4 diseases for which OntoVerbal description was available in [8] (set 4). For all 4 sets, we generated a SNOMEDtxt disease description and a concatenation of SNOMED CT content. The survey was conducted using Qualtrics survey platform (www.qualtrics.com) and included randomization: each evaluator was presented with 3 randomly selected diseases from set 1, 2 from set 2, 2 from set 3, and 1 from set 4.

In order to assess readability and general preference, we presented evaluators with SNOMEDtxt disease descriptions and the SNOMED CT content for 3 diseases from set 1 and asked whether one or the other was more readable or generally preferred, or there was no difference. Evaluators were not informed which text represented SNOMEDtxt output. Lay people found 76.5% of SNOMEDtxt disease descriptions easier to read than the SNOMED CT content, and preferred 69% of SNOMEDtxt descriptions to SNOMED CT content. Clinicians found 83% of SNOMEDtxt descriptions easier to read and preferred 44% of them to the SNOMED CT content.

We tested understanding by presenting the evaluators with either the SNOMEDtxt description or the SNOMED CT content for a concept, followed by a multiple choice question designed to test whether the evaluator understood the text; we then compared the number of correct answers given when presented with SNOMEDtxt description or with the SNOMED content. SNOMEDtxt format appeared to be significantly easier to understand for lay users: they gave the correct answer 72% of the time when presented with SNOMEDtxt description and only 51% when presented with SNOMED CT original content. There was no difference for clinicians: they gave the correct answer 100% of the time regardless of what text they were presented with.

To evaluate helpfulness, we presented evaluators with the SNOMEDtxt description, the SNOMED CT content, and a description of the same concept from either Medline Plus or Google Knowledge Graph as a reference and asked “How helpful was the terminology content compared to” the reference, on a scale from 1 to 10. Lay people found SNOMEDtxt descriptions more helpful: the average helpfulness score for SNOMEDtxt texts was 5.7, compared to 4.8 for SNOMED CT content. On the other hand, clinicians found SNOMEDtxt descriptions on average minimally less helpful than SNOMED CT content (3.50 versus 3.58).

Clinician evaluators were also asked to assess accuracy and completeness for disease concepts from set 1. In most cases clinicians thought the SNOMEDtxt descriptions were as accurate (72%) and as complete (78%) as the original content, while they found 28% of descriptions to be somewhat less accurate, 6% somewhat less complete, and 17% significantly less complete.

A conclusive comparison between OntoVerbal and SNOMEDtxt was not feasible since only 4 disease descriptions were available for OntoVerbal. We conducted a limited comparison by presenting all evaluators with the SNOMED CT content and with a disease description from either OntoVerbal or SNOMEDtxt for the same disease (evaluators were unaware of the source of each text). All evaluators were asked which text they found easier to read and generally preferred; clinicians were additionally asked whether the text description was less accurate / complete than (denoted in Table 4 as “Worse”) or as accurate / complete as (denoted as “Same”) the SNOMED CT content. This limited comparative evaluation points to a preference for SNOMEDtxt disease descriptions with the same or better performance on readability, accuracy, and completeness.

User evaluation demonstrates potential utility of SNOMEDtxt for lay users: they find the generated disease descriptions more readable and easier to understand than the structured SNOMED CT content. The accuracy and completeness of SNOMEDtxt’s natural language descriptions is close to the original SNOMED CT content. The use case of assisting in SNOMED CT content review would require some adjustments to SNOMEDtxt design in order to produce more faithful representations of the SNOMED CT content.

## Discussion

We introduce a method to generate disease descriptions directly from the SNOMED CT ontology for two main applications: providing access to definitions of rare diseases or disease variants not described in clinical reference resources and enabling easier comprehension of SNOMED CT content for those reviewing, verifying, and extending the ontology.

In the design of SNOMEDtxt, we have made several choices that favor fluidity and ease of comprehension over faithful and complete representation of information, at the risk of possible loss of information. The human evaluation of results confirms that we achieved the goal. However, these choices may not be appropriate when SNOMEDtxt output is used to verify content of SNOMED CT. It may be desirable to provide users with configurations such as “more precise” and “easier to understand” when generating the natural language texts. Another tradeoff made in the design of SNOMEDtxt was readability at the expense of generalizability. In order to extend SNOMEDtxt to other types of concepts or to other terminologies, verbalizations of relationships and handling of aggregated sentence structures would need to be adjusted.

A significant limitation to the use of SNOMEDtxt for the wider audience is the amount of content available for each disease concept in SNOMED CT. Expanding, i.e. explaining, some related nodes, for example parent disease node or finding site, may add meaningful and relevant information to the generated disease descriptions. A navigable user interface where a user could click on confusing terms and see them explained would be an alternative approach to this challenge. Developing APIs to access SNOMEDtxt would enable integration of textual disease descriptions into other electronic resources and reference materials, such as EHR help function or patient portals. The search functionality in the current implementation is limited to exact string match with either the SNOMED term name or any of the term’s synonyms and can be further improved with string search algorithms.

Results of the evaluation by lay people and clinicians presented in this paper are encouraging for the potential use of SNOMEDtxt in making SNOMED CT content more accessible and easier to review; however, a more rigorous evaluation with a larger audience and a greater number of tested concepts is recommended.

Finally, to allow the system to continuously learn and evolve, evaluation and feedback elicitation can be bulit into the user interface. Presenting users with different verbalization options at random and gathering user feedback would enable the system to learn verbalization patterns favored by users and evolve the NLG engine accordingly.

## Conclusion

This work presents an ontology verbalizer for SNOMED CT disease concepts: a tool that generates natural language concept descriptions balancing completeness and accuracy with the ease of human comprehension. User evaluation shows that lay people prefer to read natural text instead of structured ontologies and understand textual descriptions better.

More broadly, natural langauge processing is growing in importance with many potential applications in Healthcare systems. NLG involves several important tradeoffs, which should be made with a specific application in mind. Two such tradeoffs are balancing completeness and accuracy on one hand with fluidity and comprehensibility on the other; and generalizability versus linguistic polish and expressiveness.

## Figures and Tables

**Figure 3 –:**
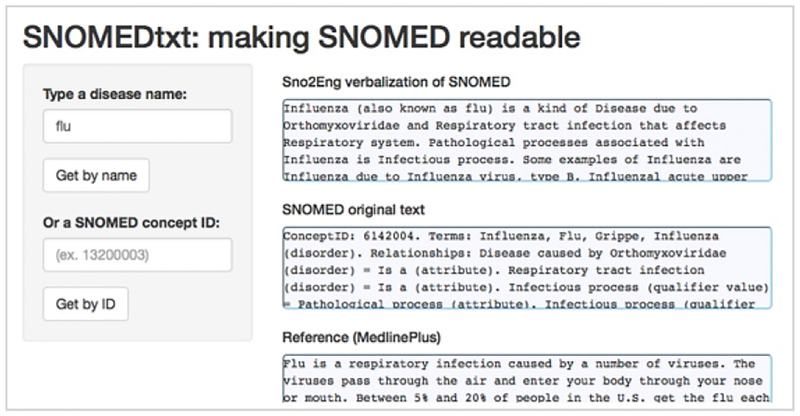
Screenshot of SNOMEDtxt Interface

**Table 1 –:** Organizing Relationships

Group	Relationship	Lexical Pattern
Definition	IS-A	“is a kind of”
	Finding site	“that affects the”
	Has definitional manifestation	“It manifests itself in”
	Associated morphology	“The associated morphology is”
	Pathological process	“Pathological process associated with … is”
	Children: IS-A, searched term=destination	“An example of … is” / “Examples of … are”
Causality	Causative agent	“is caused by”
	Due to	“occurs due to”
	Associated with	“is associated with”
Temporality	Occurrence	“presents in” (period)
	During/Following/After	“can occur during / following / after”
	Temporally related	“can be temporally related to”
Diagnosis	Finding method	“is discovered by”
	Finding informer	“<is discovered> through”
Clinical Course	Clinical course	“Clinical course is”
	Severity	“The severity of … is”
	Episodicity	“The episodicity of … is”
Other	Interprets	“interprets or evaluates”
	Has interpretation	“… as”
	Other	“Other related concepts include…”

**Table 2 –:** Evaluation with Computed Metrics

	Readability		Redundancy	
	FK	ARI	Words	Unique/All
Top 20 most searched diseases				
SNOMEDtxt	14.3	12.0	49.3	0.74
SNOMED CT	17.9	15.0	64.1	0.55
Reference	6.6	6.1	263	0.77
Random 20 SNOMED CT disease concepts				
SNOMEDtxt	11.7	9.7	47.3	0.69
SNOMED CT	15.7	13.8	69.7	0.56

**Table 3 –:** User Evaluation: SNOMEDtxt vs. SNOMED

*Readability and Preference*			
	SNOMEDtxt	SNOMED CT	No Difference
**Lay Audience (n=51)**			
Easier to read	76.5%	14.4%	9.2%
Preferred	68.6%	21.6%	9.8%
**Clinicians (n=6)**			
Easier to read	83%	11%	6%
Preferred	44%	28%	28%

*Helpfulness and Understanding*		
	SNOMEDtxt	SNOMED CT
**Lay Audience (n=51)**		
Helpful (1–10)	5.7	4.8
Correctly understood	72.1%	51%
**Clinicians (n=6)**		
Helpful (1–10)	3.50	3.58
Correctly understood	100%	100%

*Accuracy and Completeness*			
SNOMEDtxt vs. SNOMED CT	Significantly Worse	Somewhat Worse	Same
**Clinicians (n=6)**			
Accuracy	0%	28%	72%
Completeness	17%	6%	78%

**Table 4 –:** User Evaluation: Comparison with OntoVerbal

	SNOMEDtxt	Onto Verbal	SNOMED CT	No Difference
**Lay Audience (n=51)**				
Easier to read	49%	43%	3.9%	3.9%
Preferred	52%	31%	11.8%	3.9%
**Clinicians (n=6)**				
Easier to read	50%	50%	0%	0%
Preferred	50%	17%	17%	17%

	SNOMEDtxt vs. SNOMED CT		OntoVerbal vs. SNOMED CT	
**Clinicians (n=6)**	**Worse**	**Same**	**Worse**	**Same**
Accuracy	0%	45%	27%	27%
Completeness	18%	37%	18%	27%
